# Improved brachial skin hydration and appearance with hyperdiluted calcium hydroxylapatite

**DOI:** 10.1111/srt.13835

**Published:** 2024-07-18

**Authors:** Juliana Schinzari Palo, Gladstone Eustáquio de Lima Faria, Alec D. McCarthy, Ricardo Frota Boggio

**Affiliations:** ^1^ Cosmiatry department Instituto Boggio São Paulo Brazil; ^2^ Medical Affairs Merz Aesthetics Raleigh North Carolina USA

**Keywords:** CaHA, calcium hydroxylapatite, radiesse, regenerative aesthetics

## Abstract

**Introduction:**

The search for minimally invasive treatments for areas not covered by clothing, such as the arms, has increased, particularly to combat flaccidity resulting from factors such as aging and weight loss. This study evaluated the efficacy of calcium hydroxyapatite (CaHA), an injectable biostimulator, in improving flaccidity and hydration of the skin of the arms.

**Materials and Methods:**

Six women between 40 and 50 years old with visible signs of brachial flaccidity were selected. Calcium hydroxyapatite was injected into the arms in a 1:4 dilution (1.5 mL per side), with subjective evaluation based on the GAIS score and objective hydration analysis using corneometry.

**Results:**

After a single application of CaHA, there was a significant increase in skin hydration (12.2%), objectively assessed by corneometry. Patient and physician satisfaction was high, evidenced by visible improvements in photographs and by the GAIS score. No significant adverse events were reported, demonstrating the safety of the procedure.

**Discussion:**

Our clinical observations confirm the ability of CaHA to visibly improve arm flaccidity. In addition, hydration measures support previous histological studies demonstrating increases in dermal proteoglycans. Compared to other studies, the increase in skin hydration with CaHA was similar to those obtained with hyaluronic acid, suggesting comparable results with a more comfortable and less invasive technique.

**Conclusion:**

This study demonstrates the efficacy of CaHA in improving hydration of brachial skin after a single treatment. Despite the limitations of the sample size, the research contributes to the medical literature, highlighting the utility of the 3 mL CaHA presentation for brachial treatment with objective results in skin hydration.

## 1 INTRODUCTION

The increasing preference for minimally invasive esthetic interventions extends to areas of the body typically uncovered by clothing, such as the arms. The phenomenon of arm flaccidity, colloquially termed “batwing” appearance, negatively impacts individualsʼ quality of life and is exacerbated by the progressive deterioration of dermal collagen and elastin due to aging, weight loss, and UV damage.^1^, ^2^ Surgical interventions remain the benchmark for addressing extensive skin laxity in the brachial region but are associated with considerable recovery time, potential surgical complications, and scarring. For individuals with mild to moderate flaccidity, minimally invasive options offer a viable alternative with reduced adverse effects and little to no risk of cosmetically undesirable scars.

Regenerative aesthetic treatments have also grown in popularity due to their biostimulatory mechanisms of action. Notably, calcium hydroxyapatite‐carboxymethylcellulose (CaHA‐CMC; Radiesse®, Merz Aesthetics, Raleigh, NC) and poly‐L‐lactic acid (PLLA‐SCA; Sculptra®, Galerma, Dallas, TX), have demonstrated the ability to regenerate components of the dermal extracellular matrix (ECM), leading to aesthetic corrections in volume and skin quality.^3^, ^4^, ^5^ CaHA‐CMC is distinguished by its rounded, smooth, and homogeneous particles, which incite a low degrees of histological inflammation post‐application.^6^, ^7^, ^8^ The mechanism of action of CaHA‐CMC is via mechanotransduction of fibroblasts which results in the stimulation of collagen 1, collagen 3, elastin, and proteoglycans.^3^, ^9^ Importantly, it has been demonstrated that sufficient quantities of collagen and elastin are stimulated to improve skin firmness (imparted by collagen) and elasticity (imparted by elastin).^5^, ^10^ Interestingly, improvements in skin radiance have been observed following treatment with CaHA‐CMC, which could be partially explained by increases in proteoglycans.^11^ Proteoglycans are large molecules found in the ECM, composed of a core protein and one or more glycosaminoglycan chains, which are long, unbranched carbohydrates.^12^ These molecules play a crucial role in skin hydration by binding to water molecules, thereby maintaining the skin's moisture balance and providing it with a plump and hydrated appearance.^13^ A 2019 study by González and Goldberg demonstrated that treatment with CaHA‐CMC increased proteoglycan staining by an average of 76.27% in 15 subjects 6 months post‐treatment, thus signaling positive remodeling of the ECM's hydrating proteins.^14^

In 2019, Trindade et al published a consensus on the use of hyper diluted CaHA‐CMC for face and body treatment, stipulating the use of 1.5–3 mL of CaHA‐CMC per arm, per session, with a dilution of 1:2–1:4 in a diluent solution of lidocaine and saline.^15^ The rationale behind aqueous dilution of the CaHA‐CMC is based on the miscibility of CMC, the decrease in elastic modulus, and optimization of biostimulation.^9^, ^16^ Trindade et al.’s application was described as being in the subdermal plane, with linear retro injections using a cannula, with 2–4 entry points distributed on the inner side of the arms. A study by Wasylkowski assessed changes in brachial skin biomechanics following injections of CaHA‐CMC, noting improvements in patient reported arm flaccidity, skin elasticity, and dermal thickness.^10^ Another study by Mazzuco et al. confirmed these findings in a histological study, showing evidence of collagen and elastin formation and circumferential improvements in CaHA‐CMC treated arms.^17^

To date, no study has investigated the improvements in brachial skin hydration following CaHA‐CMC treatment, nor sought to objectively evaluate the hydrating correlation of increased proteoglycan content following CaHA‐CMC treatment. Therefore, based on literature demonstrating increases in dermal proteoglycans following CaHA‐CMC treatment, the aim of this study was to evaluate the clinical effect of CaHA‐CMC for improving brachial skin hydration and arm flaccidity.

## 2 MATERIALS AND METHODS

Following approval from the Research Ethics Committee (CEP) under registration number CAAE 77019124.6.0000.5492 and approval number 6.659.788, this study commenced. It included six participants aged 40–50 years who exhibited visible brachial skin flaccidity, dryness, and diminished elasticity. Exclusion criteria included previous arm surgeries, injectable treatments, energy‐based collagen stimulation procedures within six months, autoimmune diseases, current pregnancy or lactation, active skin conditions at treatment sites, or unrealistic outcome expectations.

**FIGURE 1:**
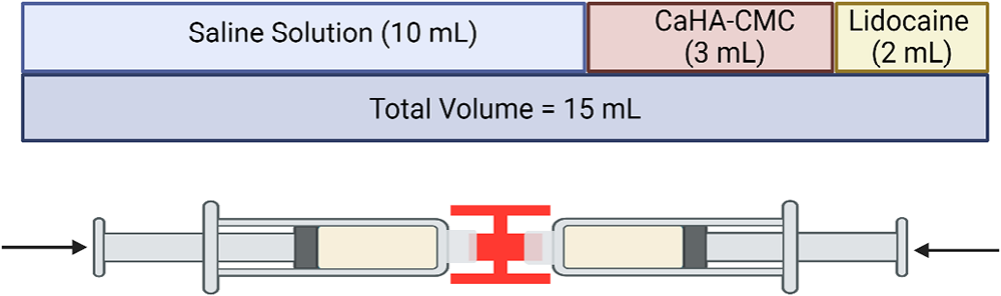
CaHA‐CMC 3 mL syringe dilution in 1:4 in 10 mL of saline and 2 mL of lidocaine with 15 mL of total volume.

Antisepsis with 2% chlorhexidine was performed before the procedure. A 3 mL Radiesse® Duo syringe (CaHA‐CMC; Merz Aesthetics, Raleigh, NC, USA) was used for treating both arms of each patient. Adhering to consensus recommendations, a 1:4 dilution ratio was used, combining 3 mL of CaHA‐CMC with 12 mL of a diluent (2 mL of 2% lidocaine and 10 mL saline) which were homogenized with at least 20 back and forth passes prior to use (Figure 1). A 22 Gauge x 50 mm cannula was used for subdermal injection of the solution via five fanning vectors, with entry points aligned with the brachial folds. Each threading vector received 1.5 mL of the solution, distributed across seven retro injection threads, totaling 7.5 mL per arm.

Before and after photographs were evaluated by an external physician using a Vectra® three‐dimensional system (Medsystems). The subjective improvement assessment was applied by both study participants and researchers, using the GAIS (Global Aesthetics Improvement Score). Hydration was assessed by a Corneometer® MPA 580 (CK Company, Cologne, Germany) that measures epidermal hydration via skin electrical capacitance after 20 min of rest in controlled ambient conditions. Follow up measurements were taken 60 days post‐intervention.

An exploratory data analysis was conducted by calculating summary measures (mean, standard deviation, minimum, and maximum) and constructing graphs. Hydration before and after the procedure was compared using Generalized Estimating Equations (GEE), as the measurements were taken in four different locations on the same volunteer (proximal and distal brachial portions of the right and left sides), that is, they present dependent measures. The adopted level and significance were set at 5%.

## 3 RESULTS

**FIGURE 2:**
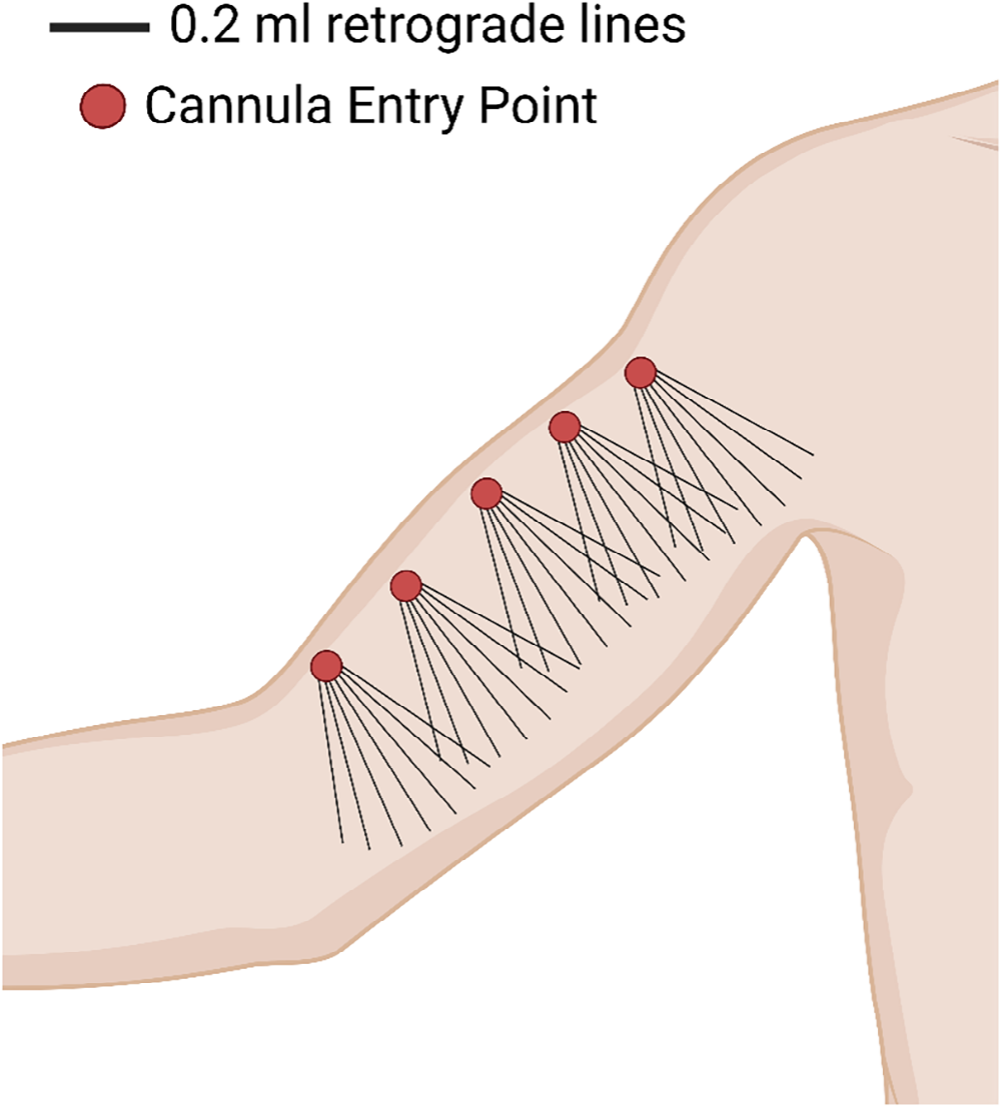
Represents the right upper limb. In red, the entry points of the cannula used for application, 22 G x 50 mm, and in black, the retroinjection lines for product deposition, with an estimated volume of 0.2 mL per retroinjection, over a length of 50 mm, resulting in a total volume of 7.5 mL per arm.

All study participants were women aged between 32 and 58 years, with an average body mass index of 24 kg/m^2^. All participants had a flaccidity degree of 2–3 according to the Amselem visual brachial flaccidity scale.^18^ The volume of CaHA‐CMC injected per arm was 1.5 mL, as recommended by Trindade et al. The 3 mL volume of CaHA‐CMC for both arms was diluted in a 12 mL solution of 2% lidocaine and saline, resulting in a final volume of 15 mL, evenly distributed across both inner sides of the arms (Figure 2). Rheologically, this dilution volume exhibits no direct filling effect and thus observed improvements are attributed to the biostimulatory effect of the CaHA‐CMC microspheres.^16^

**FIGURE 3:**
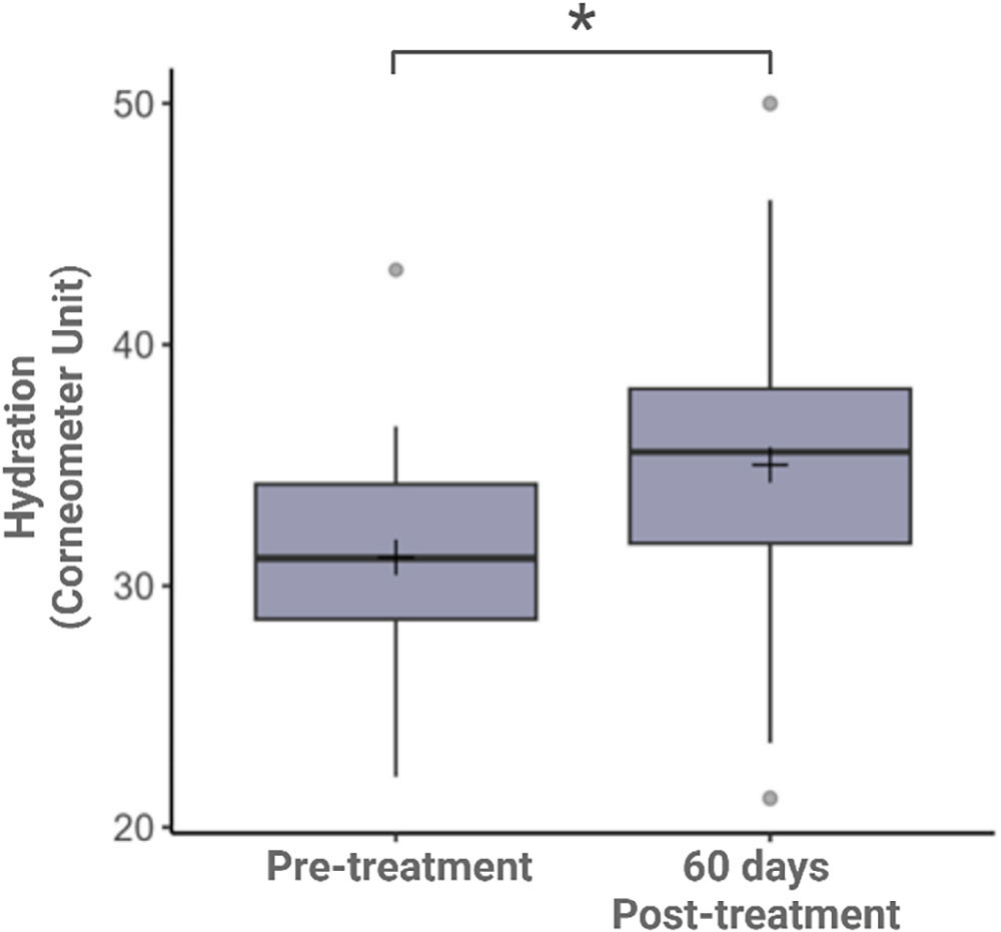
Comparative boxplot (pre‐treatment and 60 days after treatment).

**FIGURE 4:**
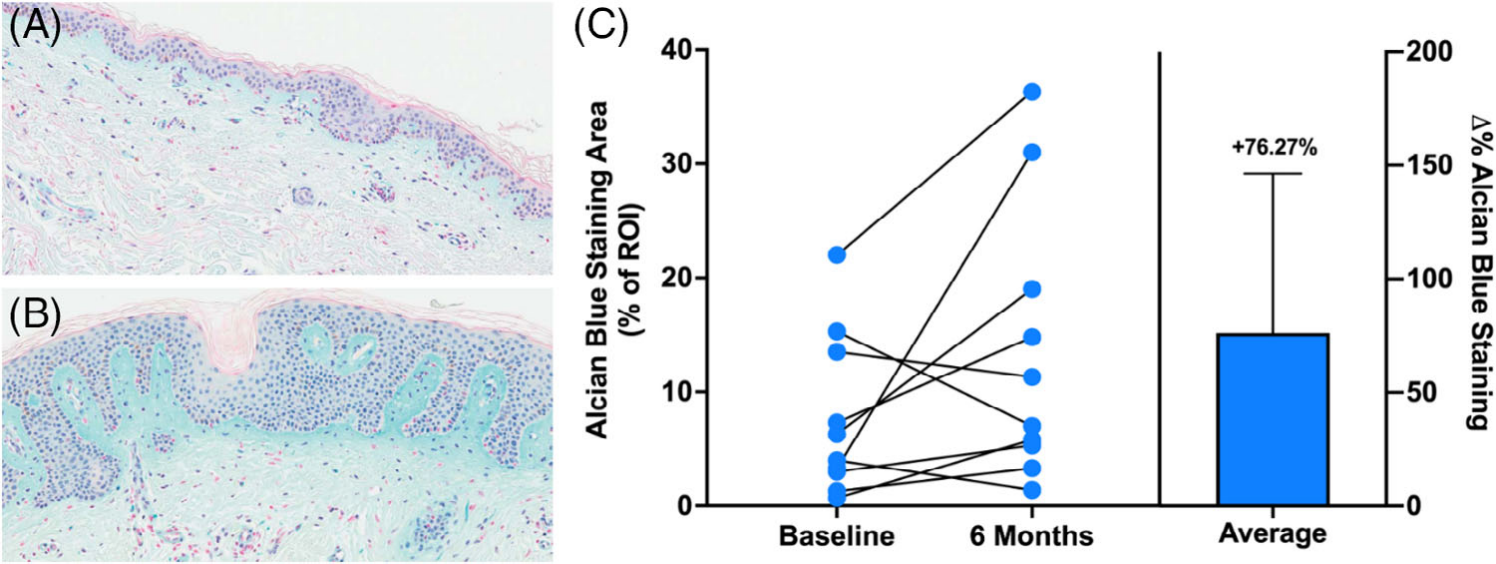
Alcian blue histological samples from (A) pretreatment and (B) 6 months post treatment with CaHA‐CMC as reported by González and Goldberg. (C) Individual changes in Alcian blue staining area and overall average change in Alcian blue staining area showing a 76.27% increase in proteoglycan‐positive areas. Images reprinted with permission from González and Goldberg (2019).^14^

Before and after treatment, patients were evaluated for skin hydration using corneometry. Corneometers® are clinically validated devices that detect changes in the electrical properties of the skin.^19^ A significant increase in hydration of 12.2% was observed after the procedure (*p*‐value = 0.016), with an average of 31.2 pre‐treatment and 35.0 post‐treatment (Table 1, Figure 3). Histological evidence of changes proteoglycans stains reported by González and Goldberg 6 months post‐injection show that 70% of patients with stain intensity values reported realized increases in proteoglycan staining, with the highest percent change being 839% and the lowest being −65% (Figure 4A–C).

**TABLE 1:** Comparison of skin hydration through corneometry between pretreatment and 60‐days post treatment.

Time	Average	Standard deviation	Minimum	Maximum	*p*‐value
Pre	31.2	4.5	22.1	43.1	0.016
Post	35.0	6.9	21.2	50.0	

**FIGURE 5:**
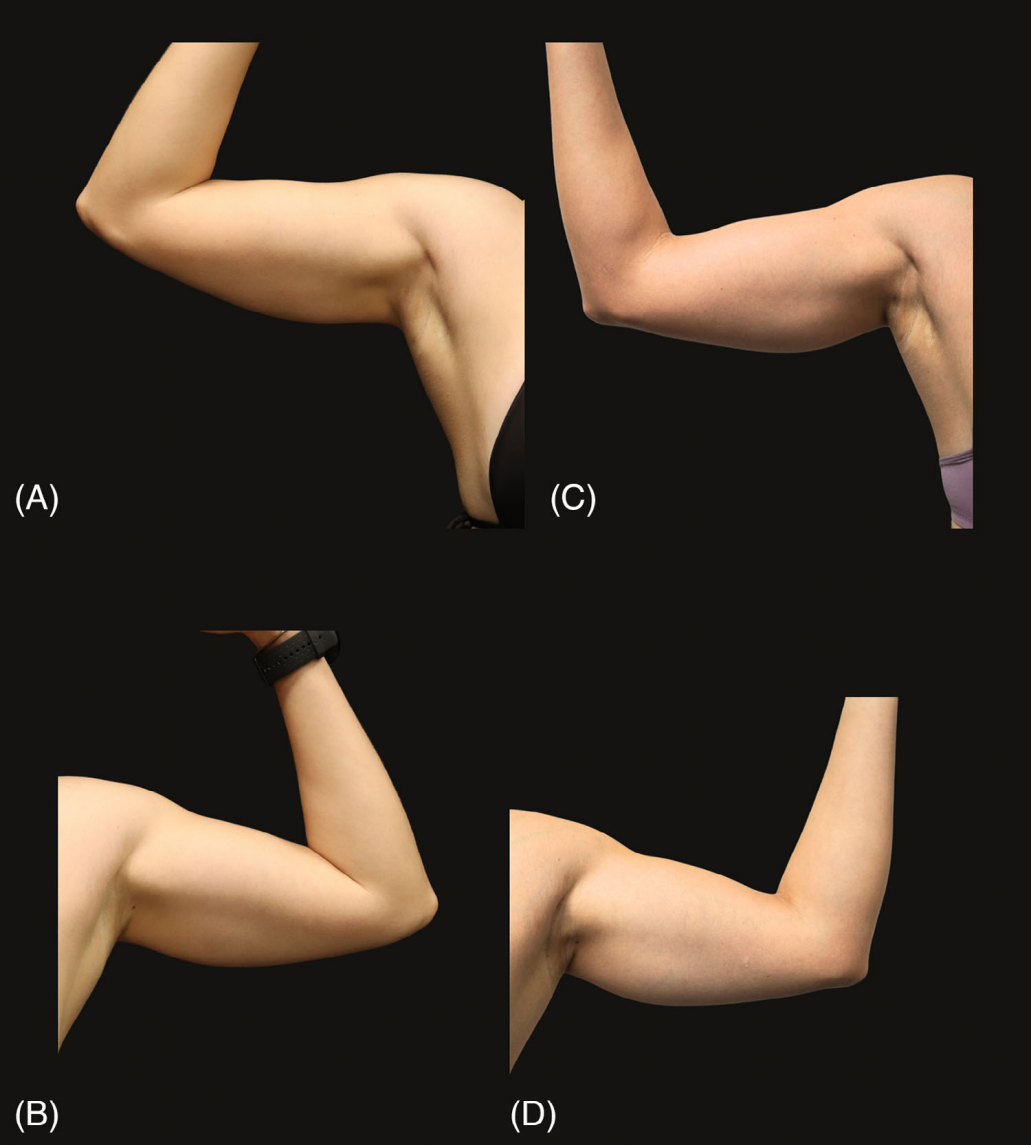
(A and B) The arms before the intervention, and (C and D), 60 days after treatment. It is possible to observe visible densification and homogeneity of the skin. In A and B, brachial laxity is noted, which is evidenced by well‐defined shading in the midline. In C and D, there is an improvement in this shading, reflecting the more turgid and firmer skin.

**FIGURE 6:**
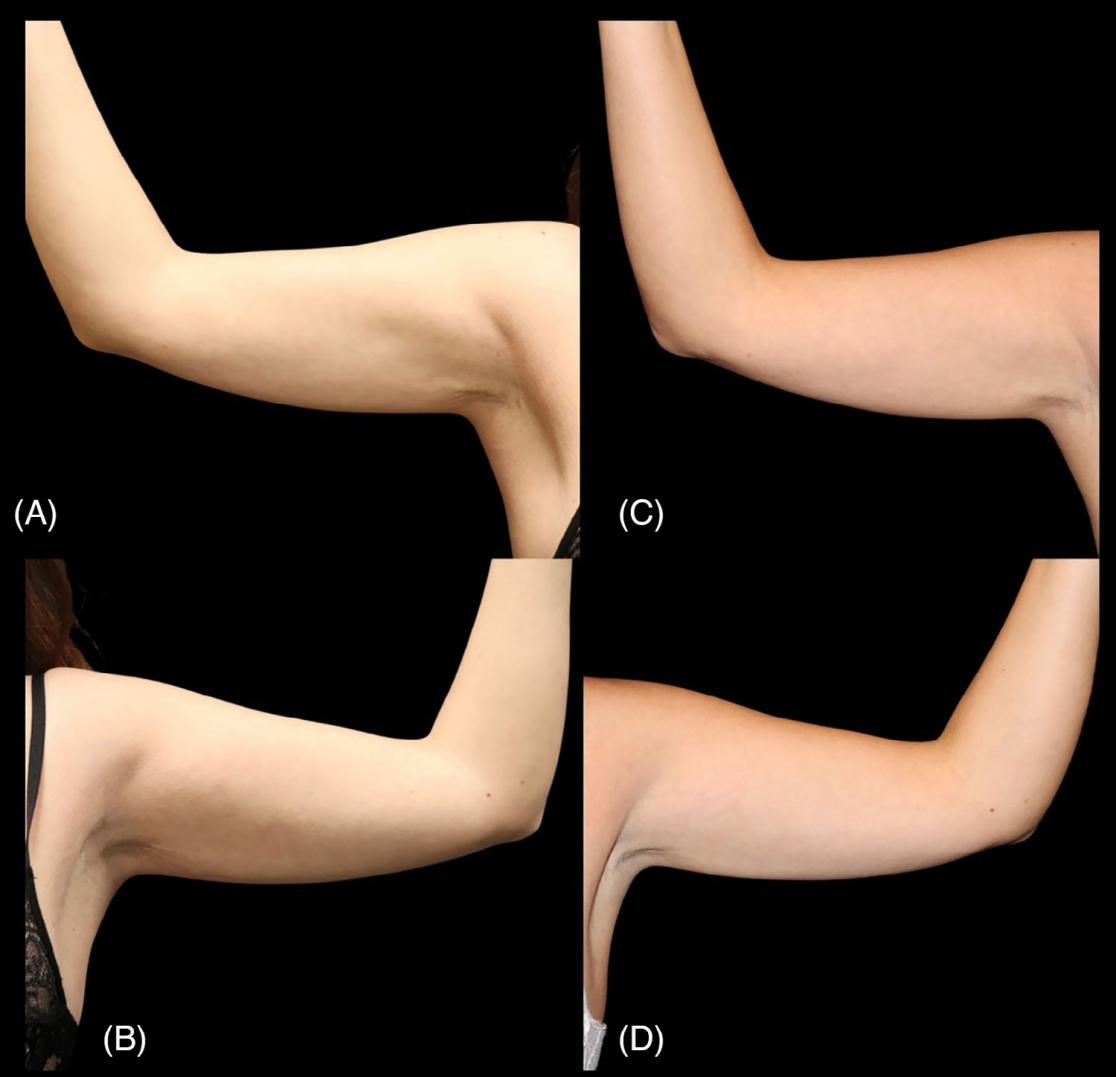
(A and B) Arms before the intervention, and C and D, 60 days after treatment. It is possible to observe visible densification and improvement of the laxity of the skin. In A and B, brachial laxity is noted, which is evidenced by well‐defined shading in the midline. In C and D, there is an improvement in this shading, reflecting the more turgid and firmer skin.

The subjective evaluation was based on the GAIS score, combined with clinical evaluation and photographic documentation of the arms before and 60 days after the treatment, by both patients and researchers. Among the involved patients, five reported significant improvement in hydration, and one observed excellent improvement. Physician grading reported that all patients showed significant improvement in skin hydration (Figures 5 and 6).

No adverse events such as nodules, pain, or significant bruising were reported. Minor bruising was observed, with spontaneous improvement, without the need for interventions. All patients reported that they would repeat the procedure or recommend it.

## 4 DISCUSSION

This research demonstrated that a singular administration of CaHA‐CMC, distributed evenly across the inner surface of the arms, significantly enhanced skin hydration and achieved high satisfaction among both clinicians and patients. A 2009 study, which explored hydration improvements using three sessions of cross‐linked hyaluronic acid (NASHA) over three months, applied a total of 6 mL of hyaluronic acid (HA), achieving a 13% hydration increase after the initial treatment and 20% post the third session, as measured by corneometry.^20^ HA is a naturally occurring disaccharide polymer that plays an important role in water retention and thus dermal hydration.^21^ However, CaHA‐CMC has no such component in its formulation, indicating the hydration effect is a result of the biostimulation of proteoglycans and endogenous GAGs.

Our findings, showcasing a 12.2% hydration boost following a single CaHA‐CMC application, align closely with the outcomes from one session of NASHA® cross‐linked hyaluronic acid. This parallel demonstrates that similar hydration levels can be attained with fewer injections and less product volume. Beyond proteoglycan stimulation, it is also conceivable that the collagen stimulation may contribute to hydration, as some studies have found that collagen fibers can absorb significant volumes of water.^22^ Visually, the improved flaccidity of the arms is attributed primarily to the stimulation of dermal elastin, which imparts the majority of the elastic effect in the skin.^23^ A post hoc histological analysis

## 5 CONCLUSION

Despite limitations such as a small sample size and the lack of a control group, this study contributes valuable insights into the effective use of CaHA‐CMC 3 mL for brachial rejuvenation. The observed increases in skin hydration are likely linked to increases in dermal proteoglycans. The visual changes in arm flaccidity are attributed to the biostimulatory properties of CaHA‐CMC, most notably the stimulation of collagen and elastin. The results from this study support the use of hyper diluted CaHA‐CMC for improving dermal hydration and improving

## CONFLICT OF INTEREST STATEMENT

Dr. Palo is a paid speaker for Profhilo® (IBSA Farmaceutici Italia, Italy). Dr. Faria is a paid speaker and trainer for Merz Aesthetics (Raleigh, NC, USA). Dr. Boggio is a paid speaker for Allergan Aesthetics, an AbbVie company (Irvine, CA, USA). Dr. McCarthy is employed by Merz Aesthetics.

## Data Availability

The data that support the findings of this study are available from the corresponding author upon reasonable request.
