# Correction to “Improved brachial skin hydration and appearance with hyperdiluted calcium hydroxylapatite”

**DOI:** 10.1111/srt.70031

**Published:** 2025-07-31

Palo JS, Lima Faria de GE, McCarthy AD, Boggio RF. Improved brachial skin hydration and appearance with hyperdiluted calcium hydroxylapatite. *Skin Res Technol*. 2024;30:e13835. doi: 10.1111/srt.13835

In the article, some text in the Discussion and Conclusion section were incomplete and inadvertently deleted.

In the Discussion section, the incomplete sentence reads:

A post hoc histological analysis

The complete sentence should read:

A post hoc histological analysis investigating elastin stimulation by Gonzalez and Goldberg verified the stimulation of dermal elastin in CaHA‐CMC treated patients, with Mazzuco et al. further confirming these findings in CaHA‐CMC treated arms.^14,17^

In the Conclusion section, the incomplete sentence reads:

The results from this study support the use of hyper diluted CaHA‐CMC for improving dermal hydration and improving.

The complete sentence should read:

The results from this study support the use of hyperdiluted CaHA‐CMC for improving dermal hydration and improving overall arm flaccidity and appearance.

We apologize for these error.

